# Supplementary material for: Spatial Logic and Evolutionary Innovation in Human Placentation
Source: bioRxiv. 2026 Sep 9:2026.09.04.749511. Preprint. [Version 1] doi: 10.64898/2026.09.04.749511 (PMC13622546; doi:10.64898/2026.09.04.749511)
Supplement: Supplementary file 1 [file media-1.pdf]

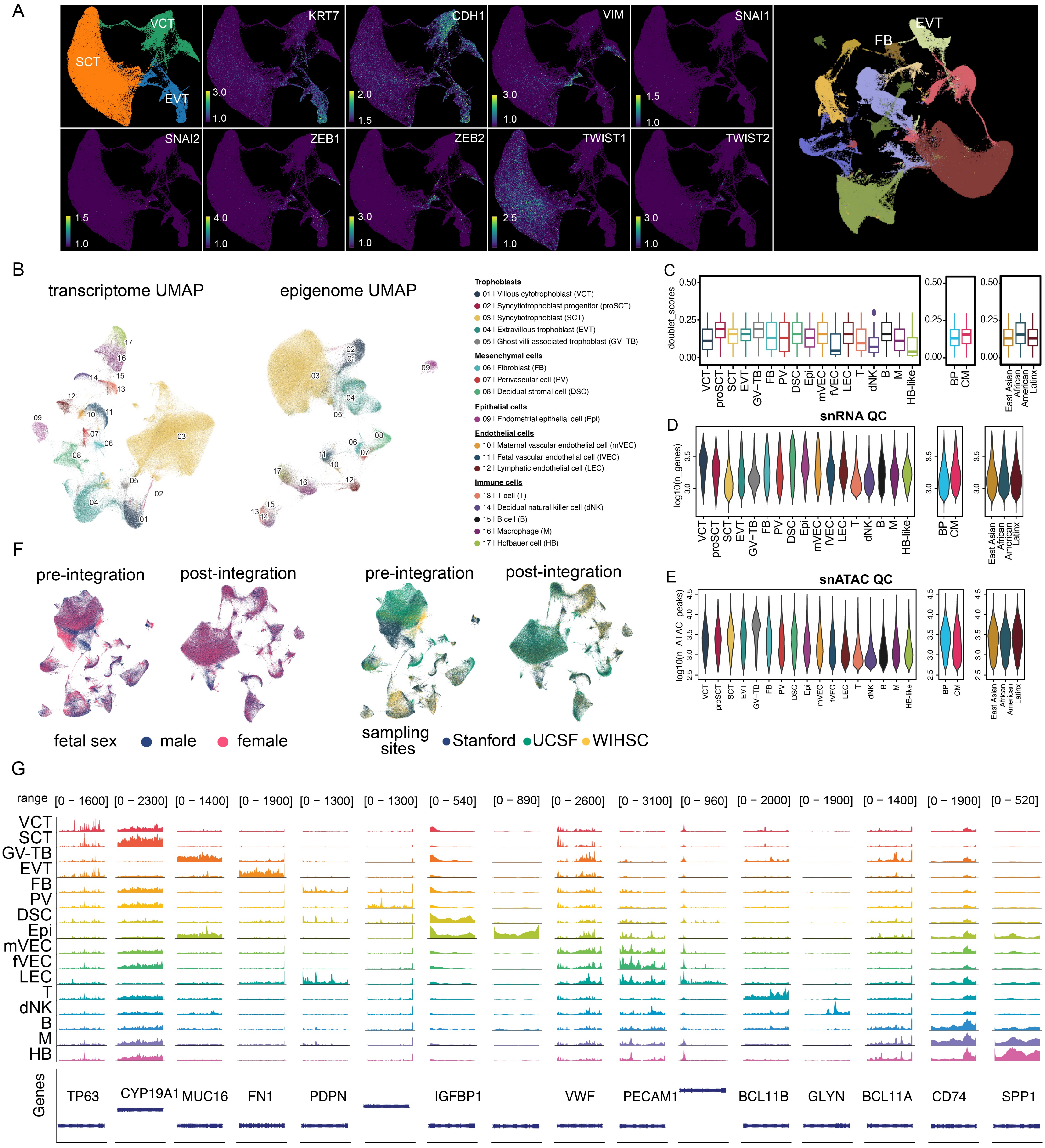
A
SCT
VCT
EVT
KRT7
CDH1
VIM
SNAI1
SNAI2
ZEB1
ZEB2
TWIST1
TWIST2
FB
B
transcriptome UMAP
epigenome UMAP
Trophoblasts
01 | Villous cytotrophoblast (VCT)
02 | Syncytiotrophoblast progenitor (proSCT)
03 | Syncytiotrophoblast (SCT)
04 | Extravillous trophoblast (EVT)
05 | Ghost villi associated trophoblast (GV−TB)
Mesenchymal cells
06 | Fibroblast (FB)
07 | Perivascular cell (PV)
08 | Decidual stromal cell (DSC)
Epithelial cells
09 | Endometrial epithelial cell (Epi)
Endothelial cells
10 | Maternal vascular endothelial cell (mVEC)
11 | Fetal vascular endothelial cell (fVEC)
12 | Lymphatic endothelial cell (LEC)
Immune cells
13 | T cell (T)
14 | Decidual natural killer cell (dNK)
15 | B cell (B)
16 | Macrophage (M)
17 | Hofbauer cell (HB)
C
doublet_scores
D
snRNA QC
log10(n_genes)
E
snATAC QC
log10(n_ATAC_peaks)
VCT proSCT SCT EVT GV−TB FB PV DSC Epi mVEC fVEC LEC T dNK B M HB-like
BP CM
East Asian African American Latinx
F
pre-integration
post-integration
fetal sex
male
female
sampling sites
Stanford
UCSF
WIHSC
G
range
[0 − 1600] [0 − 2300] [0 − 1400] [0 − 1900] [0 − 1300] [0 − 1300] [0 − 540] [0 − 890] [0 − 2600] [0 − 3100] [0 − 960] [0 − 2000] [0 − 1900] [0 − 1400] [0 − 1900] [0 − 520]
VCT SCT GV-TB EVT FB PV DSC Epi mVEC fVEC LEC T dNK B M HB
Genes
TP63 CYP19A1 MUC16 FN1 PDPN IGFBP1 VWF PECAM1 BCL11B GLYN BCL11A CD74 SPP1
