# Supplementary material for: Spatial Logic and Evolutionary Innovation in Human Placentation
Source: bioRxiv. 2026 Sep 9:2026.09.04.749511. Preprint. [Version 1] doi: 10.64898/2026.09.04.749511 (PMC13622546; doi:10.64898/2026.09.04.749511)
Supplement: Supplementary file 2 [file media-2.pdf]

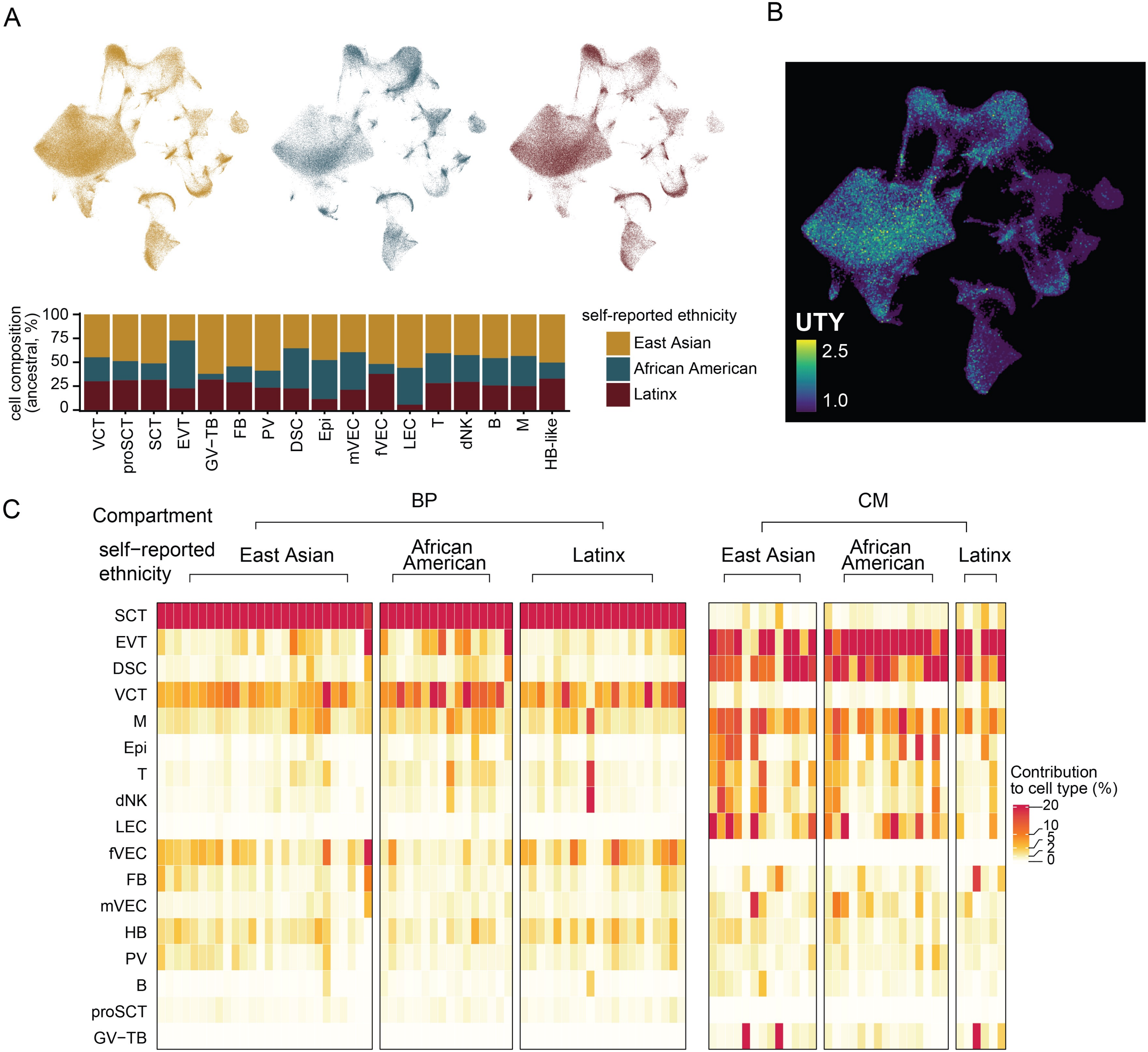

A
B
cell composition (ancestral, %)
100
75
50
25
0
VCT
proSCT
SCT
EVT
GV−TB
FB
PV
DSC
Epi
mVEC
fVEC
LEC
T
dNK
B
M
HB-like
self-reported ethnicity
East Asian
African American
Latinx
UTY
2.5
1.0
C
Compartment
BP
CM
self−reported ethnicity
East Asian
African American
Latinx
SCT
EVT
DSC
VCT
M
Epi
T
dNK
LEC
fVEC
FB
mVEC
HB
PV
B
proSCT
GV−TB
Contribution to cell type (%)
20
10
5
2
0
