# Supplementary material for: Spatial Logic and Evolutionary Innovation in Human Placentation
Source: bioRxiv. 2026 Sep 9:2026.09.04.749511. Preprint. [Version 1] doi: 10.64898/2026.09.04.749511 (PMC13622546; doi:10.64898/2026.09.04.749511)
Supplement: Supplementary file 3 [file media-3.pdf]

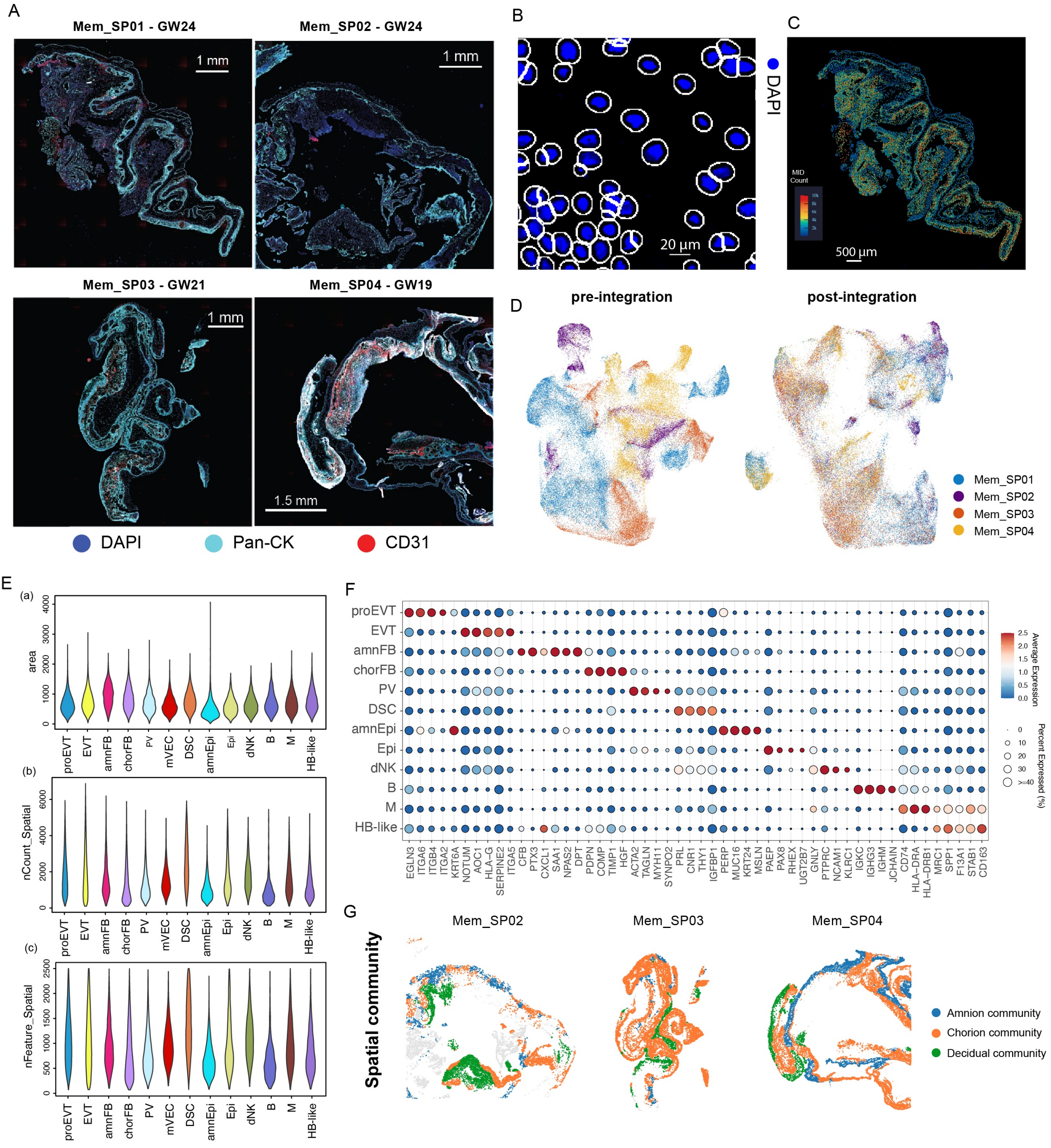

A
Mem_SP01 - GW24
Mem_SP02 - GW24
Mem_SP03 - GW21
Mem_SP04 - GW19
1 mm
1.5 mm
DAPI
Pan-CK
CD31
B
DAPI
20 μm
C
MID Count
500 μm
D
pre-integration
post-integration
Mem_SP01
Mem_SP02
Mem_SP03
Mem_SP04
E
(a)
area
(b)
nCount_Spatial
(c)
nFeature_Spatial
proEVT
EVT
amnFB
chorFB
PV
mVEC
DSC
amnEpi
Epi
dNK
B
M
HB-like
F
proEVT
EVT
amnFB
chorFB
PV
DSC
amnEpi
Epi
dNK
B
M
HB-like
EGLN3
ITGA6
ITGB4
ITGA2
KRT6A
NOTUM
AOC1
HLA-G
SERPINE2
ITGA5
CFB
PTX3
CXCL1
SAA1
NPAS2
DPT
PDPN
COMP
TIMP1
HGF
ACTA2
TAGLN
MYH11
SYNPO2
PRL
CNR1
THY1
IGFBP1
PERP
MUC16
KRT24
MSLN
PAEP
PAX8
RHEX
UGT2B7
GNLY
PTPRC
NCAM1
KLRC1
IGKC
IGHG3
IGHM
JCHAIN
CD74
HLA-DRA
HLA-DRB1
MRC1
SPP1
F13A1
STAB1
CD163
Average Expression
Percent Expressed (%)
G
Spatial community
Mem_SP02
Mem_SP03
Mem_SP04
Amnion community
Chorion community
Decidual community
