# Supplementary material for: Spatial Logic and Evolutionary Innovation in Human Placentation
Source: bioRxiv. 2026 Sep 9:2026.09.04.749511. Preprint. [Version 1] doi: 10.64898/2026.09.04.749511 (PMC13622546; doi:10.64898/2026.09.04.749511)
Supplement: Supplementary file 4 [file media-4.pdf]

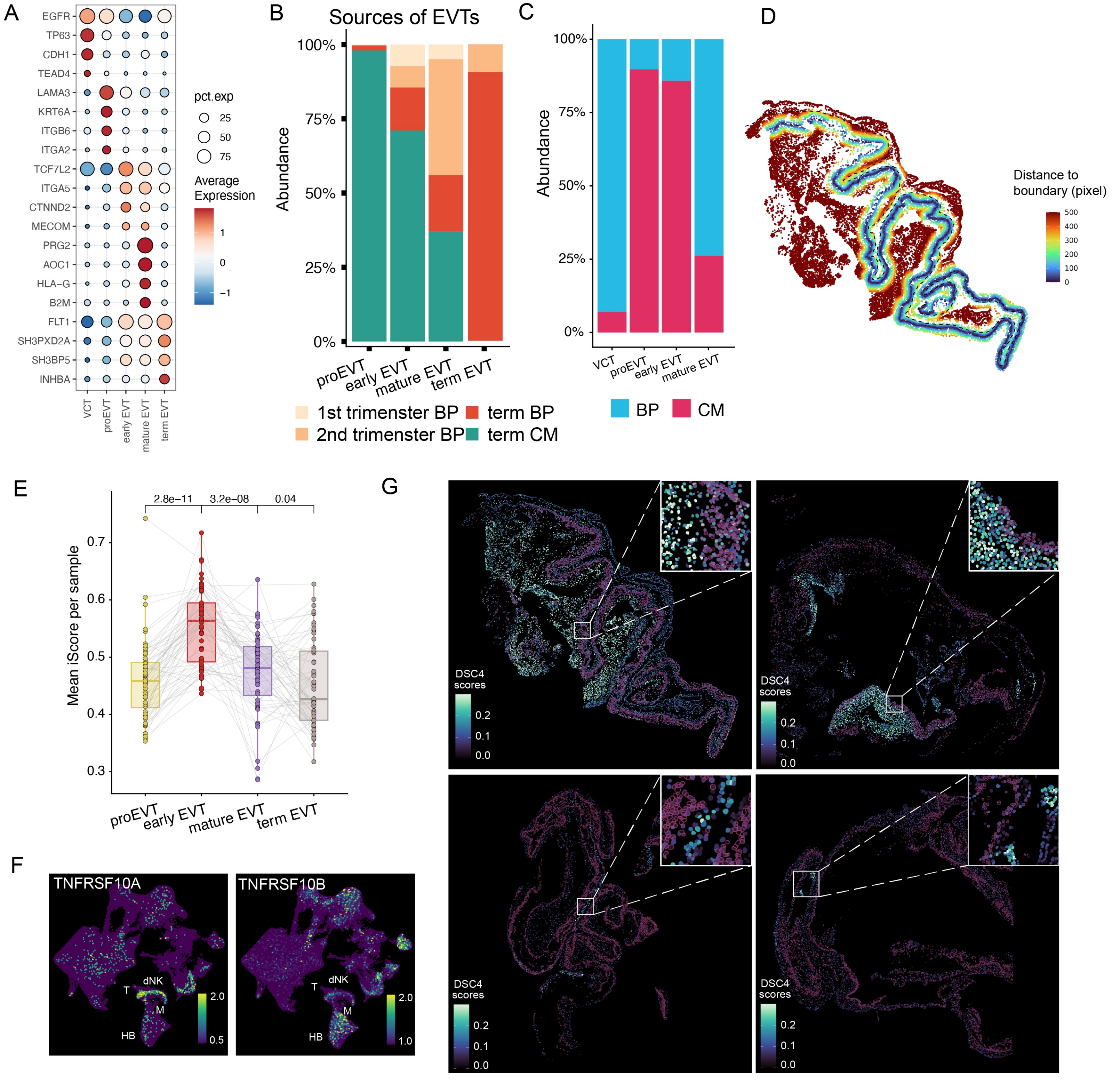

A
EGFR
TP63
CDH1
TEAD4
LAMA3
KRT6A
ITGB6
ITGA2
TCF7L2
ITGA5
CTNND2
MECOM
PRG2
AOC1
HLA-G
B2M
FLT1
SH3PXD2A
SH3BP5
INHBA
VCT
proEVT
early EVT
mature EVT
term EVT
pct.exp
25
50
75
Average Expression
1
0
−1
B
Sources of EVTs
Abundance
100%
75%
50%
25%
0%
proEVT
early EVT
mature EVT
term EVT
1st trimenster BP
term BP
2nd trimenster BP
term CM
C
Abundance
100%
75%
50%
25%
0%
VCT
proEVT
early EVT
mature EVT
BP
CM
D
Distance to boundary (pixel)
500
400
300
200
100
0
E
Mean iScore per sample
0.7
0.6
0.5
0.4
0.3
2.8e−11
3.2e−08
0.04
proEVT
early EVT
mature EVT
term EVT
F
TNFRSF10A
TNFRSF10B
T
dNK
M
HB
2.0
0.5
1.0
G
DSC4 scores
0.2
0.1
0.0
