# Supplementary material for: Spatial Logic and Evolutionary Innovation in Human Placentation
Source: bioRxiv. 2026 Sep 9:2026.09.04.749511. Preprint. [Version 1] doi: 10.64898/2026.09.04.749511 (PMC13622546; doi:10.64898/2026.09.04.749511)
Supplement: Supplementary file 5 [file media-5.pdf]

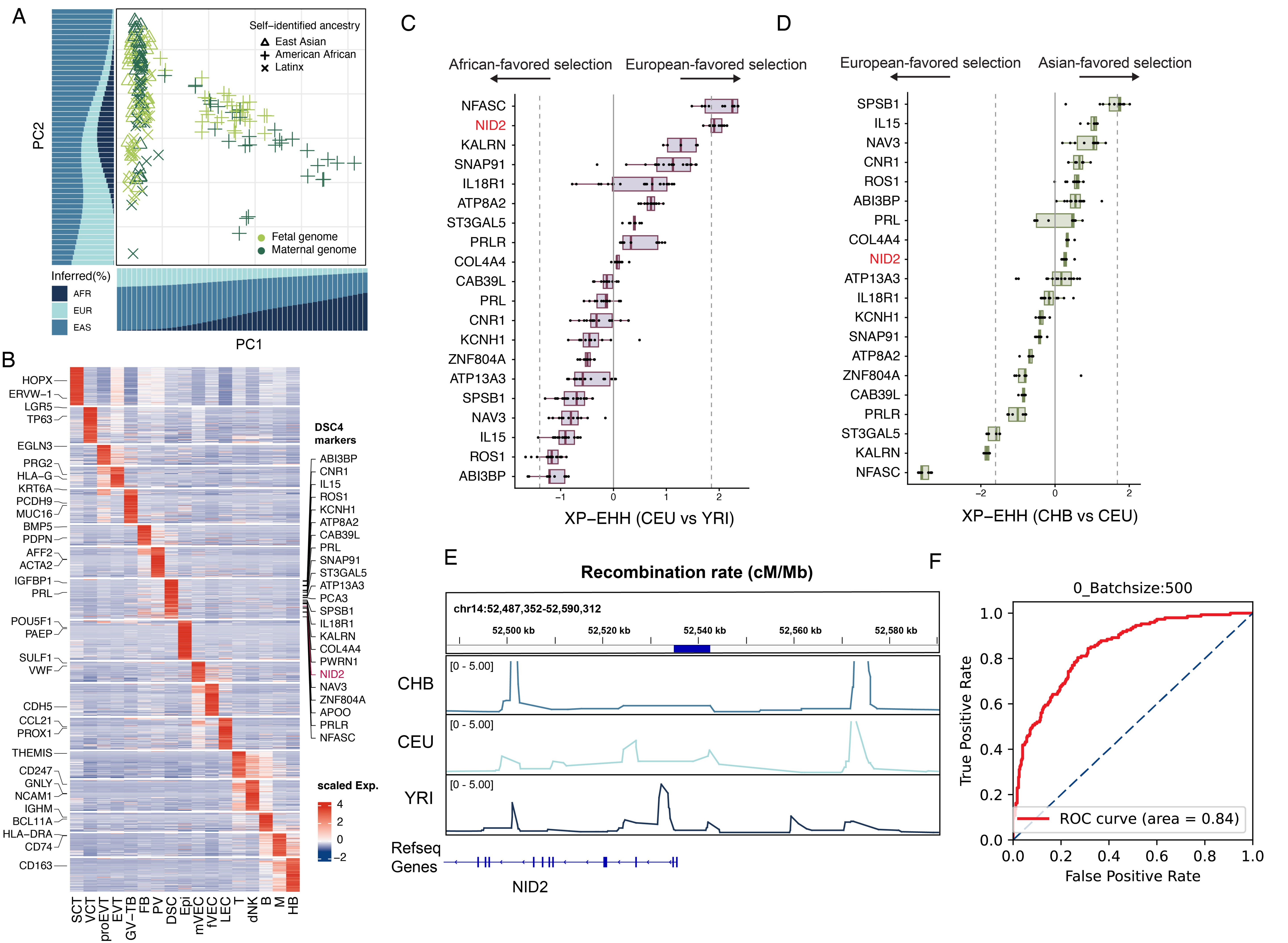

A
B
C
D
E
F
Self-identified ancestry
East Asian
American African
Latinx
Fetal genome
Maternal genome
PC1
PC2
Inferred(%)
AFR
EUR
EAS
HOPX
ERVW-1
LGR5
TP63
EGLN3
PRG2
HLA-G
KRT6A
PCDH9
MUC16
BMP5
PDPN
AFF2
ACTA2
IGFBP1
PRL
POU5F1
PAEP
SULF1
VWF
CDH5
CCL21
PROX1
THEMIS
CD247
GNLY
NCAM1
IGHM
BCL11A
HLA-DRA
CD74
CD163
SCT VCT proEVT EVT GV-TB FB PV DSC Epi mVEC fVEC LEC T dNK B M HB
DSC4 markers
ABI3BP
CNR1
IL15
ROS1
KCNH1
ATP8A2
CAB39L
PRL
SNAP91
ST3GAL5
ATP13A3
PCA3
SPSB1
IL18R1
KALRN
COL4A4
PWRN1
NID2
NAV3
ZNF804A
APOO
PRLR
NFASC
scaled Exp.
4
2
0
-2
African-favored selection
European-favored selection
NFASC
NID2
KALRN
SNAP91
IL18R1
ATP8A2
ST3GAL5
PRLR
COL4A4
CAB39L
PRL
CNR1
KCNH1
ZNF804A
ATP13A3
SPSB1
NAV3
IL15
ROS1
ABI3BP
-1 0 1 2
XP-EHH (CEU vs YRI)
European-favored selection
Asian-favored selection
SPSB1
IL15
NAV3
CNR1
ROS1
ABI3BP
PRL
COL4A4
NID2
ATP13A3
IL18R1
KCNH1
SNAP91
ATP8A2
ZNF804A
CAB39L
PRLR
ST3GAL5
KALRN
NFASC
-2 0 2
XP-EHH (CHB vs CEU)
Recombination rate (cM/Mb)
chr14:52,487,352-52,590,312
52,500 kb
52,520 kb
52,540 kb
52,560 kb
52,580 kb
CHB
CEU
YRI
[0 - 5.00]
[0 - 5.00]
[0 - 5.00]
Refseq Genes
NID2
0_Batchsize:500
True Positive Rate
False Positive Rate
ROC curve (area = 0.84)
0.0 0.2 0.4 0.6 0.8 1.0
