# Supplementary material for: Spatial Logic and Evolutionary Innovation in Human Placentation
Source: bioRxiv. 2026 Sep 9:2026.09.04.749511. Preprint. [Version 1] doi: 10.64898/2026.09.04.749511 (PMC13622546; doi:10.64898/2026.09.04.749511)

A

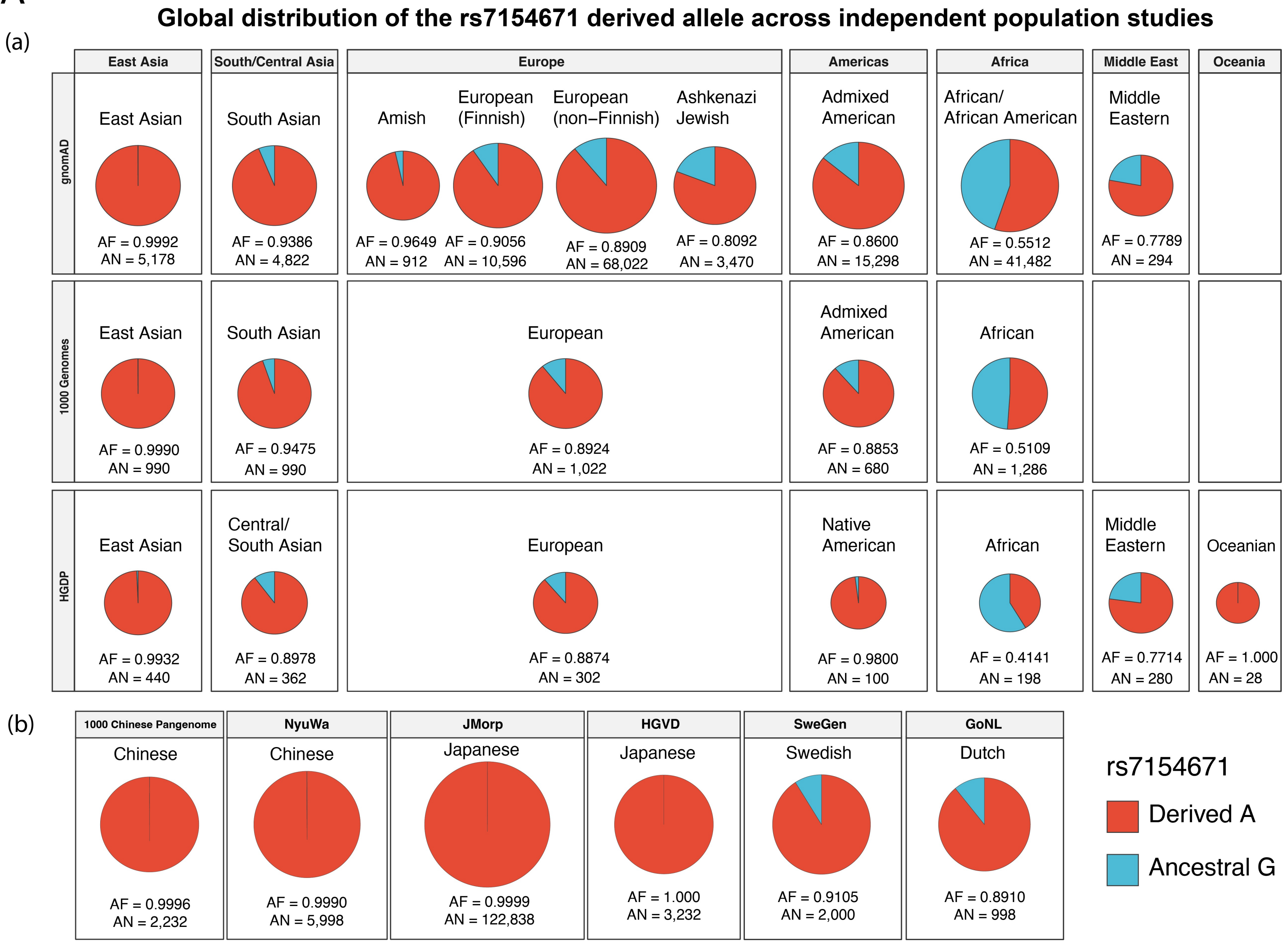

B

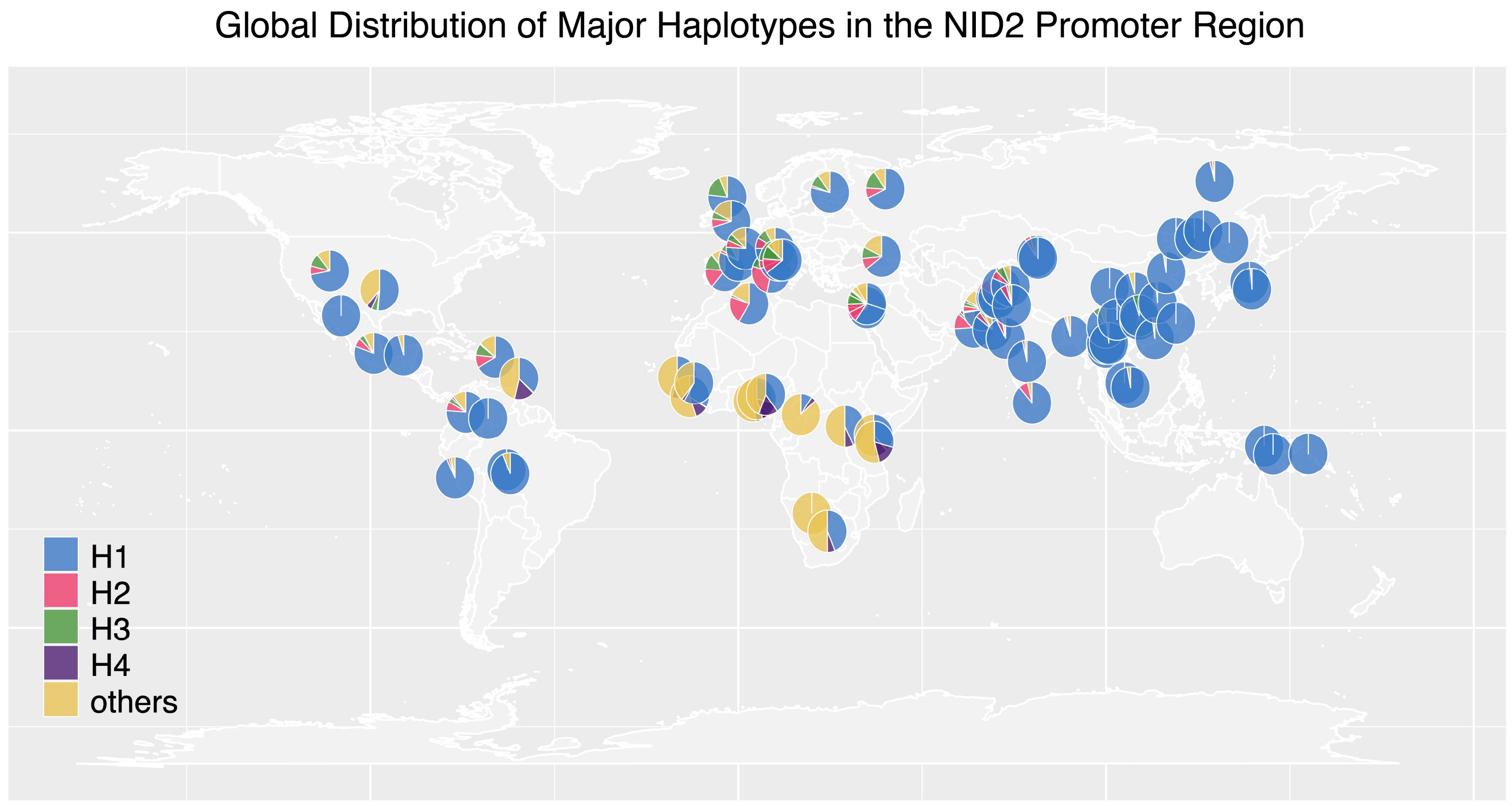

C

Sample	Lineage	Age (kya)	Region	Coverage
Ust'-Ishim	Early modern human	~45	Western Siberia, Russia	High
Ranis13	Early modern human	~45	Ranis, Germany	High
ZKU002	Early modern human	>45	Zlatý kůň, Czechia	High
Mezmaiskaya 1	Neanderthal	~60–70	Northern Caucasus, Russia	Low
Altai Neanderthal	Neanderthal	~120	Denisova Cave, Altai, Russia	High
Chagyrskaya 8	Neanderthal	~80	Chagyrskaya Cave, Altai, Russia	High
Vindija 33.19	Neanderthal	~50–52	Vindija Cave, Croatia	High
Denisova 3	Denisovan	~65–72	Denisova Cave, Altai, Russia	High

D

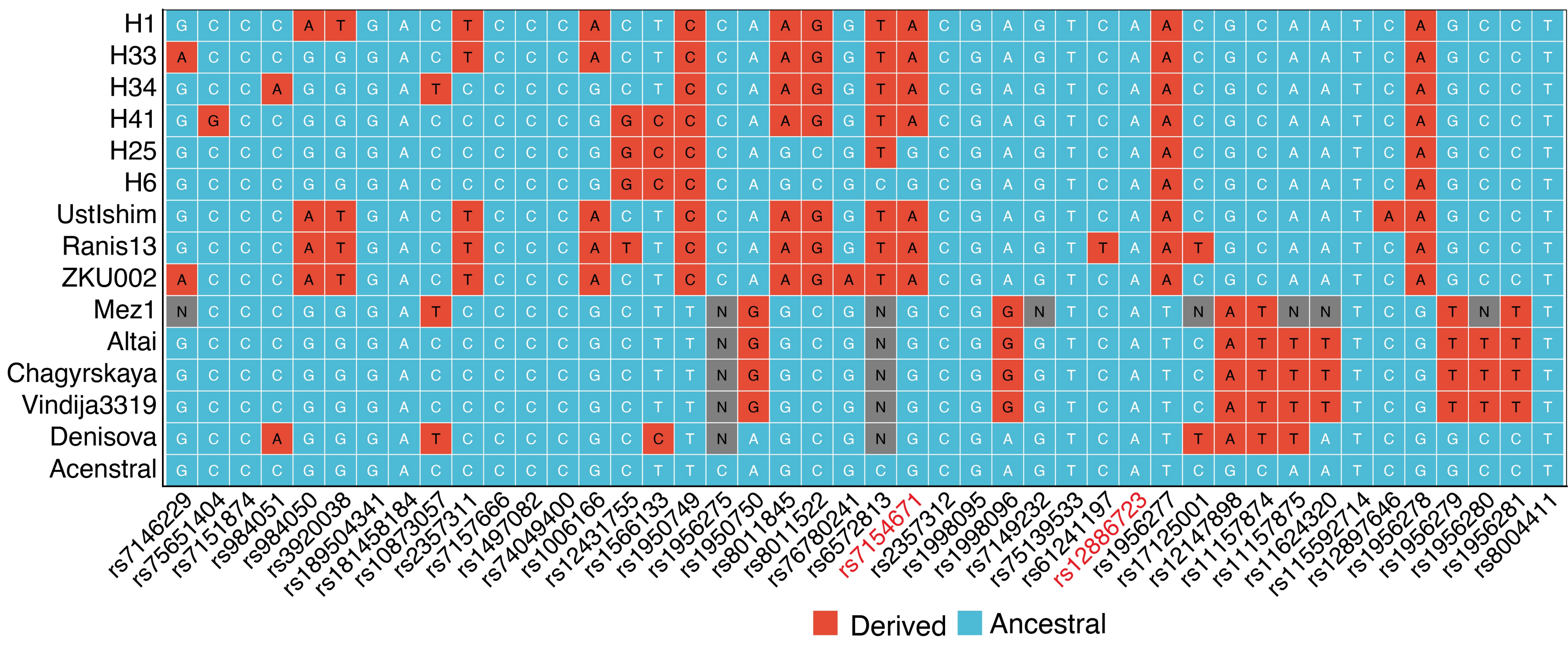

Supplement: Supplementary file 6 [file media-6.pdf]
